# Polycyclic aromatic hydrocarbons in the cestode *Oncomegas wageneri* parasite of Mexican flounder *Cyclopsetta chittendeni*

**DOI:** 10.1007/s00436-019-06597-z

**Published:** 2020-02-01

**Authors:** Lilia C. Soler-Jiménez, Emanuel Hernández-Núñez, Iván Velázquez-Abunader, Arturo Centeno-Chalé, Víctor M. Vidal-Martínez

**Affiliations:** Laboratorios de Parasitología y Pesquerías, Centro de Investigación y de Estudios Avanzados del IPN (CINVESTAV-IPN) Unidad Mérida, Carretera Antigua a Progreso Km. 6, Cordemex, C.P. 97310 Mérida, Yucatán Mexico

**Keywords:** Contamination, Parasite, *Oncomegas wageneri*, Flatfish, Bioaccumulation, Bioindicator

## Abstract

The concentrations of polycyclic aromatic hydrocarbon metabolites (PAHm) and their bioconcentration factors (BCF) were determined in the larval stages of the cestode *Oncomegas wageneri*, recovered from the intestine of the Mexican flounder *Cyclopsetta chittendeni*, in the southern Gulf of Mexico. The PAHm concentrations in *O. wageneri* were measured using fixed-wavelength fluorescence spectrometry and compared with PAHm concentrations in host bile. *Oncomegas wageneri* PAHm concentrations were markedly higher than those in host tissues. The highest BCF values were obtained for 1-hydroxypyrene (OHP) and benzo(a)pyrene (BaP). Using a General Linear Model, a significant negative relationship was found between *O. wageneri* PAHm concentrations (as response variable) and the number of *O. wageneri* and oil well proximity. Low BCF values and PAHm concentrations in *C. chittendeni* correlated positively with *O. wageneri* PAHm concentrations. In contrast, high BCF values for PAHm concentrations in *C. chittendeni* had a negative association with *O. wageneri* PAHm concentrations. This study provides the first evidence of the presence of PAHm in intestinal larval cestodes of marine flatfishes, demonstrating levels of PAHm that were higher than levels in their hosts.

**Electronic supplementary material:**

The online version of this article (10.1007/s00436-019-06597-z) contains supplementary material, which is available to authorized users.

## Introduction

Marine fish can acquire polycyclic aromatic hydrocarbons (PAHs) from exposure to oil extraction sediments or oil spills (Pérez-del-Olmo et al. 2007; Pérez-del-Olmo et al. 2009; Centeno-Chalé et al. 2015). Other PAH sources are continental, such as products of the incomplete combustion of wood, coat, or even from barbecued meat (Baek et al. 1991; Motorykin et al. 2015). These PAHs with terrestrial origin are transported by rivers or air to the sea (Baek et al. 1991; Morrison and Boyd 1998). In the southern Gulf of Mexico, coastal and offshore petroleum extraction is a significant economic activity (García-Cuellar et al. 2004; Vidal-Martínez et al. 2019) that, together with natural hydrocarbon release from oil seeps, exposes marine organisms to hydrocarbons. Additionally, processed hydrocarbons, such as motor oils, diesel, and lubricants, together with other contaminants, such as heavy metals, pesticides, and fertilizers (e.g., N and P in different compounds), are also transported from the continent and released in marine waters (Morrison and Boyd 1998). Consequently, benthic organisms (such as flatfishes and their parasites) are likely to be exposed to a wide variety of both inorganic and organic pollutants, including PAHs. However, research on the accumulation of organic compounds in host–parasite systems is scant (e.g., Heinonen et al. 1999, 2000; Persson et al. 2007; Brázová et al. 2012a; Oluoch-Otiego et al. 2016), especially about hydrocarbons, compared with studies on parasite–metal interactions (Tenora et al. 2000; Pascual and Abollo 2003; Sures et al. 2006; Barus et al. 2007; Johnson-Restrepo et al. 2008; Palm 2011; Brázová et al. 2012b; Lim and Shin 2013; Zahra et al. 2013; Brázová et al. 2015; Kleinertz et al. 2016). Difficulty in accessing crude oil for experimental tests limits studies to the occurrence of accidental oil spills (e.g., Pérez-del-Olmo et al. 2007; Pérez-del-Olmo et al. 2009; Centeno-Chalé et al. 2015), compounding the lack of understanding regarding the bioaccumulation of hydrocarbons in host–parasite systems. The literature suggests a fractionation of organic compounds between parasites and their hosts related to the commonly observed bioaccumulation pattern of organic chemicals, i.e., lipophilic (represented by the octanol–water partition coefficient, K_ow_) substances that are stored in lipids (Sures 2004). Generally, parasites have lower lipid contents than their hosts. Thus, the accumulation level of lipophilic substances is expected to be lower in parasites than in the host tissues (Heinonen et al. 1999, 2000). Despite this general trend, chemical fractionation in host–parasite systems can vary depending on the physiological and behavioral processes of the parasites.

When exposed, marine fish and other aquatic vertebrates have the capacity to metabolize PAH and produce excretion metabolites that are detectable in the bile (Lin et al. 1996; Aas and Klungsøyrb 1998; Aas et al. 2000; Beyer et al. 2010). Invertebrates, such as polychaetes, can also metabolize PAH and produce excretion metabolites (Tairova et al. 2009). In addition to the other techniques, the preliminary detection of these metabolites can be performed by simple and affordable techniques, such as fixed-wavelength fluorescence spectrometry (Beyer et al. 2010). As suggested by Beyer et al. (2010), even when this technique cannot distinguish between closely related PAH isomers, it can distinguish fish bile metabolites from PAH contaminated and uncontaminated sites.

Parasites can also produce metabolites as a reaction to organic compounds. For example, when larval cestodes, such as *Schistocephalus solidus* (a fish parasite), *Echinococcus granulosus*, and *Taenia solium* (human parasites), are exposed to organic compounds, such as 1,2-dichloro-4-nitrobenzene or 1,2-epoxy-3-(p-nitrophenoxy) propane, they produce specific metabolites through the glutathione transferase (GST) system (Torres-Rivera and Landa 2008 and references therein). Furthermore *E. granulosus* and *T. solium* are also able to use detoxification enzymes, such as thioredoxin, glutathione peroxidase, or thioredoxin glutathione reductase against organic compounds (Tsai et al. 2013; Wang et al. 2018). Here, we examine the levels of PAH metabolites in larval cestodes relative to their marine flatfish hosts using fixed-wavelength fluorescence spectrometry.

*Oncomegas wageneri* is a larval cestode infecting flatfishes collected from oil extraction zones of the Mexican Gulf of Mexico (Vidal-Martínez et al. 2014; Centeno-Chalé et al. 2015; Vidal-Martínez et al. 2015; Martínez-Aquino et al. 2019). This parasite is a good candidate for the detection of PAH metabolites because it has a wide geographical distribution in the Gulf of Mexico, has a high prevalence and mean abundance in its host, and inhabits the intestine of the flatfishes where it can be exposed to whatever the host is ingesting (including food contaminated with PAH) (Vidal-Martínez et al. 2014). Support for this suggestion comes from the findings of Oluoch-Otiego et al. (2016), who have shown that intestinal cestodes bioaccumulate higher levels of polychlorinated biphenyls (PCBs) than their hosts. Additionally, *O. wageneri* is relatively easy to collect and identify from fish dissections.

Therefore, this parasite–host system is considered to be a potential good model for determining whether *O. wageneri* could accumulate higher concentrations of polycyclic aromatic hydrocarbon metabolites (PAHm) compared with its host, the Mexican flounder *Cyclopsetta chittendeni*. In this study, fixed-wavelength fluorescence spectrometry was used for the following two reasons: (1) this technique is widely used around the world to detect production of PAHm in fish bile samples (Beyer et al. 2010; Pampanin et al. 2016) and (2) fixed-wavelength fluorescence spectrometry is a rapid and cost-effective technique, which is ideal to the exploratory nature of this study in trying to determine whether the larval cestodes could accumulate higher concentrations of polycyclic aromatic hydrocarbon metabolites (PAHm) than their hosts. Therefore, the aim of the present study was to determine and compare the concentrations of PAHm in intestinal larval stages of *O. wageneri* with those of their host the Mexican flounder, *C. chittendeni*, using fixed-wavelength fluorescence spectrometry.

## Materials and methods

### Collection and examination of fish

**Fig. 1:**
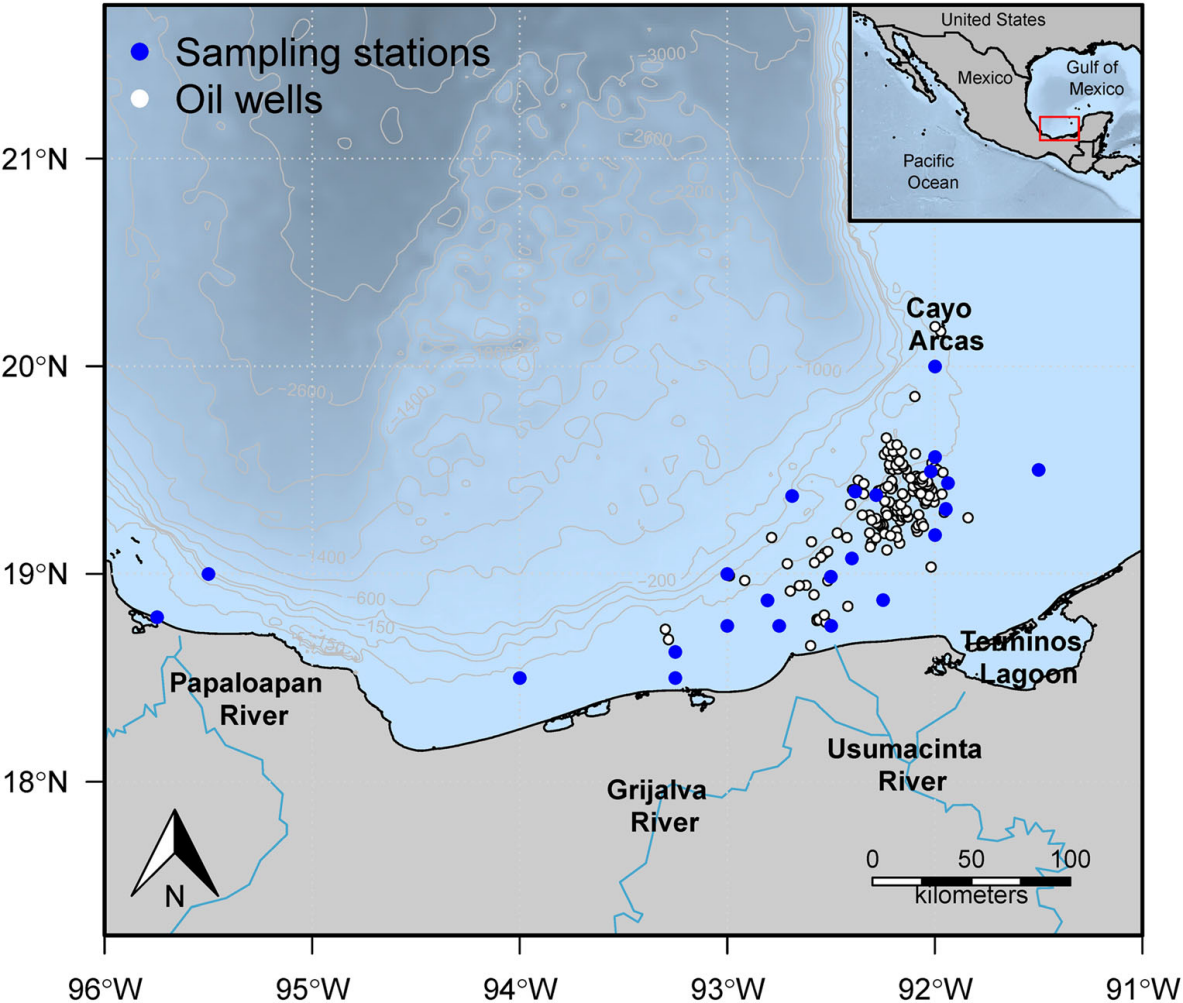
Sampling stations and oil wells within the Campeche Sound, southern Gulf of Mexico

The study area included 34 sampling stations in the southern Gulf of Mexico (Fig. 1). Samples were obtained from August to October 2015 at depths of between 10 and 175 m, using shrimp trawl nets. The trawls lasted 50–60 min at 0.6–0.7 knots around each station. Samples were obtained from the oceanographic vessel (OV) Justo Sierra. A total of 55 Mexican flounders, *C. chittendeni*, were collected, deep frozen (− 20 °C) in individual plastic bags, and transported to CINVESTAV-IPN Mérida Unit for parasitological examination. The total length (cm) and weight (g) were recorded for each fish. Subsequently, the body surface, cavities, and all internal organs of each fish were examined for metazoan parasites using a dissection microscope. All the parasites found of different taxa were counted in situ and divided into two groups. The first group, which included all the parasites (i.e., digeneans, nematodes, and acanthocephalans) except *O. wageneri*, was preserved in 70% alcohol and used for both molecular and morphological taxonomy (see Vidal-Martínez et al. (2014) for details). In contrast, the second group, which included the *O. wageneri* larvae, was frozen at −20 °C immediately after extraction from the fish without any solution. Therefore, the larvae of *O. wageneri* used for the PAH analysis were never in contact with 70% alcohol. At the analysis time, the larvae of *O. wageneri* were washed in ultrapure water and placed directly in 1 mL of 1:1 (*v*/*v*) solution of methanol (spectrophotometric grade) and molecular-grade water (working solution), for the determination of PAHs.

### Analytical procedures

All *O. wageneri* individuals were washed in ultrapure water and analyzed for the presence of PAHm. Fixed-wavelength fluorescence spectrometry was used to measure PAHm rather than individual compounds (Beyer et al. 2010). Four PAHm families were analyzed, each having a different number of benzene rings: benzo(a)pyrene (BaP) for hydrocarbon with five benzene rings; 1-hydroxypyrene (OHP) for hydrocarbon with four rings; phenanthrene (Phe) for hydrocarbons with three rings; and 2-naphthol (Naph) for hydrocarbon with two rings. All measurements were performed with a Thermo Scientific Lumina Fluorescence spectrofluorometer and 1-cm quartz cells. Spectral data acquisition and processing were carried out with the use of Luminous V.3.0 software.

For the extraction of PAHm, 0.01 g of *O. wageneri* individuals was homogenized using a manual tissue homogenizer (TissueRuptor Qiagen) in 1 mL of a 1:1 (*v*/*v*) solution of methanol (spectrophotometric grade) and molecular-grade water (working solution). The homogenized samples were centrifuged at 4700*g* for 10 min (Centrifuge Thermo Scientific Micro CL 17) to separate the solid and liquid phases. Subsequently, 25 μL aliquots of liquid phase (containing organics compounds) were placed in 1-cm quartz cells with 2 mL of working solution for spectrofluorometric measurement. The procedure was applied also to bile samples from Mexican flounders. Four calibration curves, with six points each, were built using the following four standard solutions: 0.63–3.11 ng mL^−1^ for benzo[a]pyrene, 0.55–2.71 ng mL^−1^ for 1-hydroxypyrene, 44.38–219.75 ng mL^−1^ for phenanthrene, and 35.95–177.78 ng mL^−1^ for 2-naphtol (Supplementary Material, Fig. S1). The calibration curves with their corresponding correlation coefficient (*R*) and determination coefficient (*R*^2^) were calculated by least-square linear regression analysis. The limits of detection and quantification were calculated from the calibration curves, using the following equations: LOD = 3.3_S0_ and LOQ = 10_S0_ (ICH 2005), where LOD corresponds to the limit of detection; LOQ, the limit of quantification; and S0, the standard deviation of the blank (Bruce 1999). The standard curves were used to estimate the approximate concentration of PAHm in unknown samples. Table 1 shows the coefficients of correlation (*R*) and determination (*R*^2^), confirming the linearity of the method with values of *R* > 0.99 for all PAHs determining the region of the curve where there is a direct relation between the instrumental response and the concentration of the analyte. The specific wavelength conditions for each compound required the calibration curves to be created separately; samples were analyzed at each of the wavelength conditions. An analysis of variance refuted the null hypothesis of equal variances, so the homogeneity of variances was assessed with ANOVA regression test, because it is not sensitive to the violation of this assumption (Supplementary Material, Tables S1 to S4).

**Table 1:** Parameters for equations of each calibration curve

Compounds	Excitation (nm)	Emission (nm)	Rings	Range (ng/mL)	LOD (ng/mL)	LOQ (ng/mL)	*Α*	*β*	*R*	*R*^2^
Benzo(a)pyrene	364	404	5	0.63–3.11	0.003	0.008	34.90	3971.00	0.99	0.99
1-Hydroxypyrene	348	386	4	0.55–2.71	0.001	0.003	612.95	7654.50	0.99	0.99
Phenanthrene	363	392	3	44.38–219.75	0.943	2.859	67.02	88.01	0.99	0.99
2-Naphthol	308	470	2	35.95–177.78	1.738	5.267	427.95	47.23	0.99	0.98

nm, nanometers; LOD, limit of detection; LOQ, limit of quantification; *A*, intercept value; *β*, slope; *R*^2^*,* coefficient of determination

In the Supplementary Material (Tables S5 to S8), levels 1, 2, and 3 represent the percentage of recovery and the relative standard deviation, the values of which meet the acceptance criteria of 100% ± 25% and relative standard deviation variation between 0.7 to 15% for fourth-group PAHs (EURACHEM/CITAC 2003).

### Data analysis

Bioconcentration factors (BCFs) for PAHm were determined, based on Sures et al. (1999), where the ratio of PAHm concentration in the parasite relative to that in host bile (BCF = C_(parasite)_ / C_(host bile)_) was calculated. In addition, differences between total PAHm concentrations (∑PAHm) and those of individual PAHm in parasites and in fish bile were tested for statistical significance using the Kruskal–Wallis non-parametric analysis with multiple comparisons of mean ranks for all samples and Dunn’s test for post-hoc multiple comparisons (Zar 1999). The significance of all statistical analyses was established at *α* = 0.05 unless otherwise stated. SigmaPlot 11 software was used for the statistical analyses.

Additionally, the geostatistical kriging method (Cressie 1993) was used to determine the existence of spatial patterns of total PAHm concentration in parasites and hosts. This method calculates the average weights based on the observed values, as well as their spatial trends through variogram functions (Bellier et al. 2007). Four models were tested (spherical, cubic, Gaussian, and Matern) to determine which variogram best described the spatial pattern of PAHm concentrations. PAHm values were transformed to log (PAHm). The best model was selected with the Akaike information criterion (AIC), as proposed by Burnham and Anderson (2002). The geostatistical analyses were conducted using the geoR package (Ribeiro and Diggle 2016) with R programming language (R Core Team 2017).

Exploratory data analysis was carried out to evaluate parasite PAHm concentration behavior with respect to six independent variables (number of individual parasites, fish total weight, individual parasite weight, PAHm concentration in host, BCF, and proximity to oil extraction wells). For this analysis, a Pearson’s correlation matrix was used to test for collinearity. A value of *R* ≥ 0.7 was established as a decision criterion for discarding those variables with collinearity problems (Zar 1999; Cárdenas-Palomo et al. 2015). The link function (*η* = *μ*) was selected considering the following two criteria: highest deviance explained and lowest AIC value (Burnham and Anderson 2002). A stepwise selection model was used to select the independent variables to be included in the final model. All procedures were performed using glm function of the base package of R programming language (R Core Team 2017).

To determine the potential effect of the six independent variables on the total PAHm concentrations in parasite (the dependent or response variable), a generalized lineal model (GLM) was used (Nelder and Wedderburn 1972). These models have the flexibility of adding quadratic or higher-order effects to improve the explanation of the response variable.

Because there is no background about the possible distribution that could be followed by the total PAHm concentration in parasites, three probability distributions were tested (Gaussian, gamma, and inverse Gaussian). As a result of the previous analysis, the Gaussian distribution was chosen because the error of the fitted model was getting closer to *ɛ* ~ *N* (0, *σ*^2^) (Zar 1999).

## Results

**Fig. 2:**
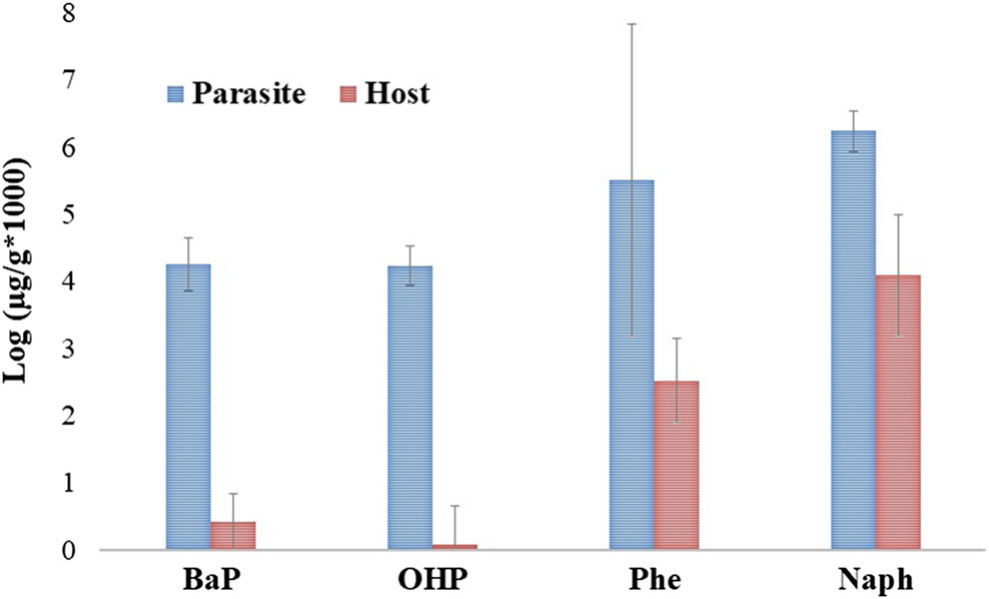
PAHm concentrations in *Oncomegas wageneri* and *Cyclopsetta chittendeni* bile. BaP, benzo(a)pyrene; OHP, 1-hydroxypyrene; Phe, phenanthrene; Naph, 2-naphthol

A total of 55 *O. wageneri* pools obtained from 55 *C. chittendeni* from 34 sampling stations were used for comparison of PAHm concentrations. The mean PAHm concentrations for *O. wageneri* ($\overline{x}=$2139.33 ± 2664.20 μg/g) were found to be significantly higher than those of their hosts ($\overline{x}=$12.91 ± 32.10 μg/g) (*P* < 0.05). Significant differences between *O. wageneri* and fish bile were confirmed for total PAHm concentrations (*H* = 81.8; *P* < 0.05) and for each PAHm concentration, showing significantly higher values in parasites in all cases (BaP H = 7.1, *P* < 0.05; OHP H = 8.246, *P* < 0.05; Phe H = 5.17, *P* < 0.05; and Naph H = 7.1, *P* < 0.05) (Fig. 2). Comparison between each PAHm concentration in *O. wageneri* and fish bile separately showed that naphthol concentration values were significantly higher (*P* < 0.05) in both parasites and hosts compared with all other PAHm measured. BaP and OHP presented the lowest values (Fig. 2). The BCF ratios indicated that parasites had higher PAHm concentrations than those of their host tissues. The highest BCF values were obtained for 1-hydroxypyrene (OHP) (14,597.9) and benzo(a)pyrene (BaP) (6957.5), compared with those obtained for phenanthrene (Phe) (1003.1) and 2-naphthol (Naph) (140.8).

**Fig. 3:**
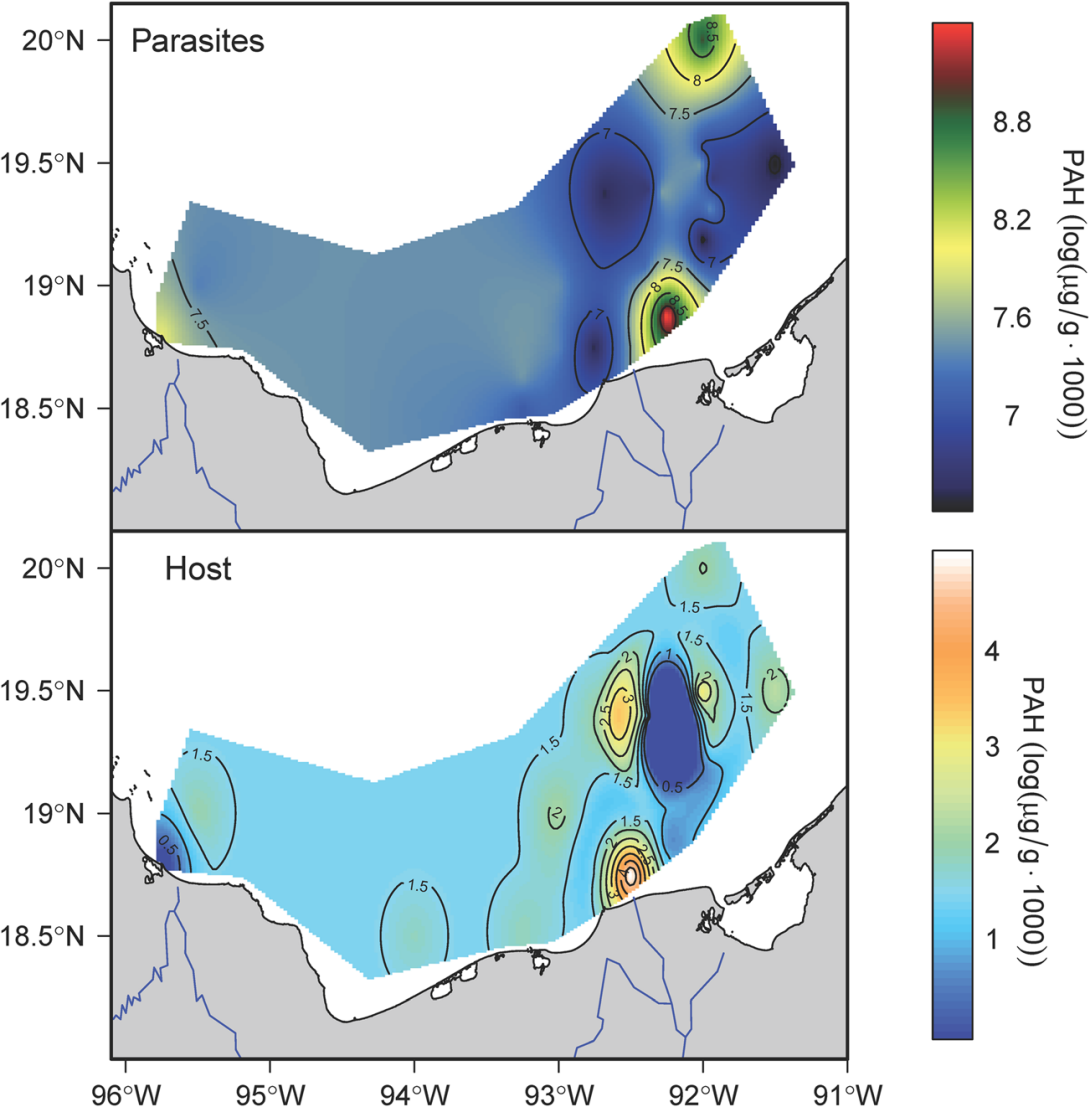
Spatial distribution patterns of total PAHm concentrations in *Oncomegas wageneri* (parasites) and *Cyclopsetta chittendeni* (host). Red areas on maps represent a high mean PAHm concentration for the organisms sampled at each station, whereas blue zones represent a low mean PAHm concentration

The spatial distribution pattern for total PAHm concentration in *O. wageneri* and *C. chittendeni* in the study area is shown in Fig. 3. High total PAHm concentrations in parasites were found just off the Campeche coast and near Cayo Arcas. For the hosts, total PAHm concentrations were high on the Campeche coast.

### General linear model

**Fig. 4:**
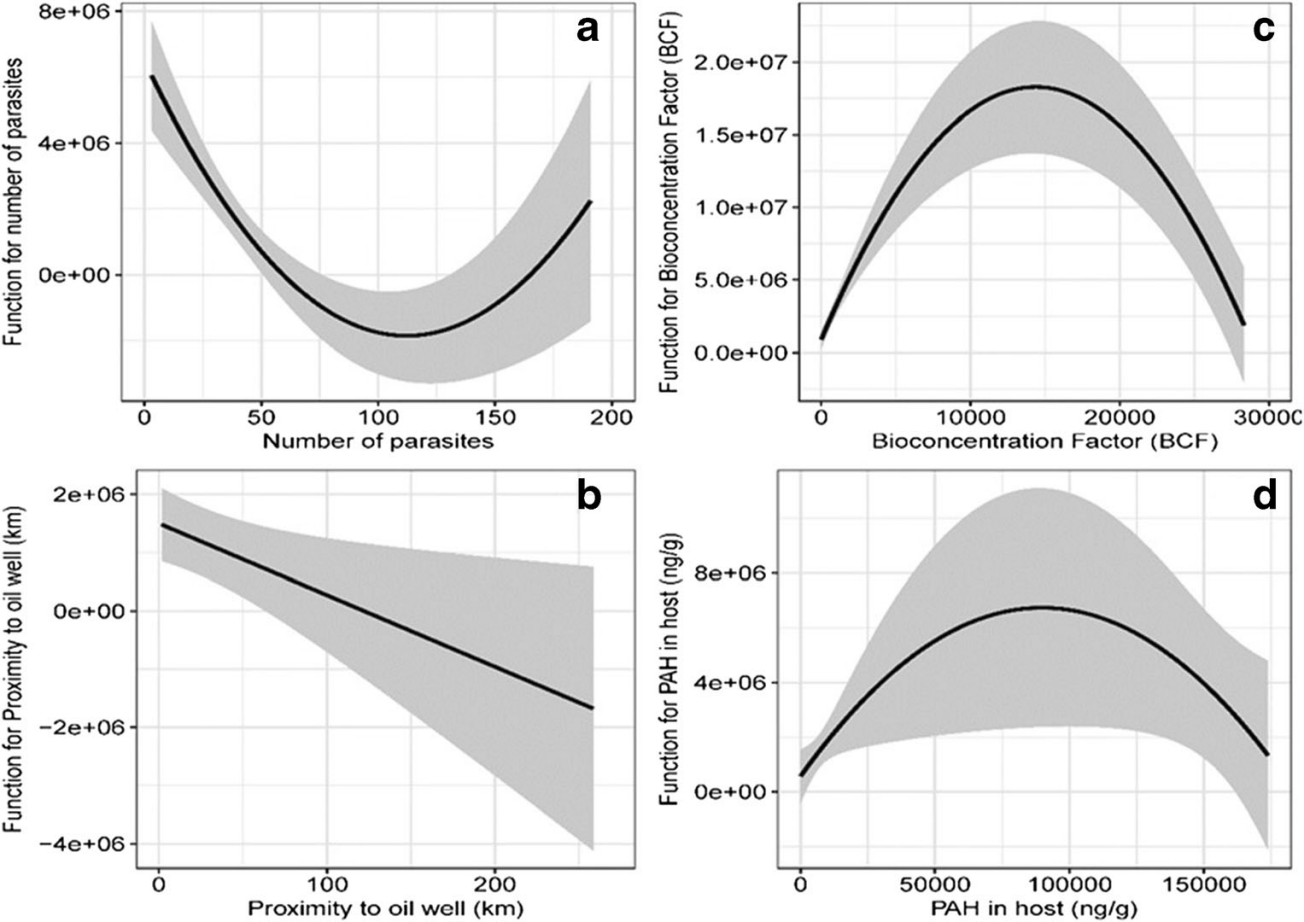
Fit plots for general linear models (GLM) that show the effect of the four independent variables retained by the GLM model on the total parasite PAHm concentration as the dependent variable

Of the six variables originally included in the GLM model to explain the behavior of parasite total PAHm concentrations, only four were retained. These variables were number of individual parasites, proximity to oil wells, BCF, and host total PAH concentrations (Table 2). The final model explained 60% of deviance, in most cases showing a non-linear relationship with the dependent variable (Fig. 4). With increase in the number of individual parasites, there was a concave up behavior, whereas, with proximity to oil wells, there was a linear decrease in the parasite total PAH concentration with increase in the distance from the oil wells (Fig. 4A and B). The behavior of BFC and host total PAHm concentration was also non-linear. In both cases, the function had a concave down shape, initially presenting a positive effect on parasite total PAHm concentration, followed by an inflection point and a decrease in the dependent variable with increase in the values of the independent variables (Fig. 4C and D).

**Table 2:** Coefficients of the best general linear model (GLM), with parasite PAHm concentration as dependent variable and four independent variables. The asterisk indicates statistical significance of the coefficient (*P* < 0.05). Values of the Akaike information criterion (AIC) of the distributions tested for the Generalized Lineal Model (GLM) are shown in Table S9

Variable	Coefficient	Standard error of coefficient	*t* value	*P* value
Intercept	5.193e+06	1.015e+06	5.118	5.650e−06*
Number of parasites	− 1.486e+05	2.628e+04	− 5.655	8.930e−07*
BCF	2.422e+03	3.312e+02	7.313	2.740e−09*
Proximity to oil well	− 1.231e+04	5.106e+03	− 2.410	0.0199*
PAHm in host	1.375e+02	6.065e+01	2.267	0.0281*

## Discussion

This study provides the first evidence to our knowledge of the capacity of the larval parasite *O. wageneri* to accumulate and probably produce PAH metabolites, highlighting the role of this fish parasite as an indicator of bioaccumulation compared with that of their hosts. More importantly, the results showed that the larval cestode, *O. wageneri*, accumulated PAHm in higher concentrations than their fish host, *C. chittendeni*.

The higher accumulation capacity of *O. wageneri* compared with *C. chittendeni* tissues was also confirmed for BCFs obtained for all four hydrocarbon metabolites. Several studies have demonstrated that some parasite groups, such as adult acanthocephalans, cestodes, and some nematodes, are able to bioaccumulate both organic and inorganic compounds in higher concentrations than their hosts (Tenora et al. 2000; Heinonen et al. 2001; Sures and Siddall 2001, 2003; Pascual and Abollo 2003; Sures 2003; Sures and Reimann 2003; Sures et al. 2006; Barus et al. 2007; Azmat et al. 2008; Jankovská et al. 2012; Brázová et al. 2012a; Brázová et al. 2012b; Amini et al. 2013; Nachev et al. 2013; Zahra et al. 2013; Brázová et al. 2015; Nachev and Sures 2016; Kleinertz et al. 2016; Oluoch-Otiego et al. 2016); even the presence of some parasitic organisms may influence positively their hosts (Brázová et al. 2012a). Brázová et al. (2012a) found that the acanthocephalan *Acanthocephalus lucii* attached to the intestine of perch absorbed significantly higher concentrations of polychlorinated biphenyls (PCBs) than the muscles, liver, kidney, brain, and adipose tissue of their host. In infected perch, PCB levels in the liver and muscle were about 20 times lower and 3 times lower, respectively.

The higher concentration of PAHm in parasites with respect to their host has at least the following four possible explanations: (1) *O. wageneri* absorb PAHm from the host and do not excrete them as efficiently as their host; (2) high numbers of *O. wageneri* individuals interfere with absorption of the compounds by the host, affecting PAHm concentrations in both parasite and host; (3) that the PAHm bioconcentration in *O. wageneri* depends on the partition coefficient (K_ow_) of each hydrocarbons, because higher BCFs were found for PAH with higher number of rings; (4) *O. wageneri* individuals produce PAHm metabolites in higher concentrations than their hosts. In our view, based on the available information, the most probable explanations are (1), (2), and (3). However, explanation (4) cannot be discarded. Even though it is likely that *O. wageneri* acquires PAH metabolites from *C. chittendeni*, the possibility that the parasite produces its own PAH metabolites cannot be ruled out. Clearly, to determine whether or not *O. wageneri* produces its own PAH metabolites requires further research.

The first explanation for the higher concentration of PAHm in parasites with respect to their host is that *O. wageneri* individuals absorb PAHm from the host but do not excrete them efficiently. Owing to the lack of a digestive system, the substances enter the cestodes across the tegument; therefore, the cestodes ingest and absorb the same type of food acquired by the host (Nachev et al. 2017; Sures et al. 2017), a characteristic that places *O. wageneri* in the same trophic level of the host. For such host–parasite systems, no significant difference between pollutant concentrations in the hosts and the parasites would be expected (see Le et al. 2014). However, our results showed a higher concentration of PAHm in *O. wageneri* with respect to the Mexican flounder. Therefore, the most likely explanation is that *O. wageneri* does not eliminate these PAHm as efficiently as the fish and accumulates these compounds. For vertebrates, such as fish, once the PAH are in the body, they are metabolized by the enzymes of the superfamily of cytochrome P450 (CYP) to more water-soluble hydroxy-PAH (OH-PAH) (Motorykin et al. 2015). For many years, it was suggested that oxidative metabolism was not a significant process in parasitic helminths, and cytochrome P450 (CYP) activity was generally absent or occurred at a very low level (Pemberton and Barrett 1989; Precious and Barrett 1989; Barrett 1998, 2009; Torres-Rivera and Landa 2008; Yadav et al. 2010). However, it has been shown that larval cestodes, such as *S. solidus* (a fish parasite), *E. granulosus*, and *T. solium*, (human parasites) are able to produce specific metabolites through the glutathione transferase (GST) system in response to the exposure to organic compounds, such as 1,2-dichloro-4-nitrobenzene or 1,2-epoxy-3-(p-nitrophenoxy) propane (Torres-Rivera and Landa 2008 and references therein). Furthermore, *E. granulosus* and *T. solium* are also able to use detoxification enzymes, such as thioredoxin, glutathione peroxidase, or thioredoxin glutathione reductase, against organic compounds (Tsai et al. 2013; Wang et al. 2018). Additionally, CYP oxidase presence has been demonstrated in some parasitic nematodes (Kotze 1997; Cvilink et al. 2008; Laing et al. 2015). For example, the genome of *Caenorhabditis elegans* encodes 80 CYPs (Menzel et al. 2001, 2005). Cvilink et al. (2008) showed that CYP activity is higher in the larval stages than in adult stages of *Haemonchus contortus*, which reflects an increased metabolic activity in response to exposure of free-living stages to environmental toxins. All this bring us to suggest that *O. wageneri* would be eliminating PAHm but not as efficiently as their host, finally acting as a sinkhole of PAHm. Similar arguments for parasites acting as sinkholes of heavy metals have been provided by Nachev et al. (2017) and Sures et al. (2017).

Our second explanation was that high numbers of *O. wageneri* individuals could be interfering with the absorption of compounds by the host, which in turn would be affecting PAHm concentrations in both parasites and host. Apparently, this is the case since our results revealed significant negative associations between PAHm concentrations in parasites and the intensity of infection. A biodilution effect can explain this pattern. The biodilution theory has been confirmed through laboratory experiments and synoptic studies of multiple ecosystems, where the biological accumulation of several compounds depends on the biomass found (Chen and Folt 2005). For example, Brázová et al. (2015) recorded that a high intensity of infection was consistently associated with low heavy metal concentration in *Acanthocephalus lucii* and *Proteocephalus percae*. Likewise, Heinonen et al. (2000) found that uninfected clams *Pisidium amnicum* accumulated benzo(a)pyrene (BaP) and 2,4,5-trichlorophenol at significantly higher levels than clams infected with trematodes. Indeed, our results concur with the general trend reported by other authors with respect to the decrease in pollutant concentration in the hosts with increase in the number of parasites (Sures and Siddall 2003).

Our third explanation was that the PAHm bioconcentration in *O. wageneri* depended of the partition coefficient, because higher BCFs were found for PAH with higher number of rings. First, the PAHm concentrations in parasites were at least four orders of magnitude higher than those in the hosts. Second, the ranking order of BCFs (OHP > BaP > Phe > Naph) suggests that the high molecular weight PAH, namely OHP and BaP, even when they had the lowest concentrations in both parasites and hosts (Fig. 2) were retained at higher proportions in parasites than in their hosts. This result suggested that the parasites were unable to eliminate these high molecular weight PAHm compared with the fish hosts, which contained very low levels of these hydrocarbons. A possible reason is that aromatic compounds of high molecular weight are more resistant to the mechanisms of degradation owing to the number of benzene rings that they have (4–5), in addition to the energy required to break these bonds (Abdel-Shafy and Mansour 2016). In this case, *O. wageneri* apparently would not be able to remove or excrete this type of high molecular weight PAHm. In contrast, the BCFs for Naph and Phe were lower than those for OHP and BaP, which indicates that both parasites and hosts are able to remove these low molecular weight PAHm, but certainly at a greater extent in the host than in the parasite (Fig. 2). This pattern is also reflected in the results of the GLM analysis (Fig. 4). Under these conditions, we infer a possible PAHm removal process by *O. wageneri* which is related to the chemical structure and molecular weight of each compound. Therefore, the bioaccumulation not only involves bioavailability and absorption potential, but it is also strongly influenced by chemical compound-specific properties, such as lipophilicity (represented by the octanol–water partition coefficient, K_ow_), which is closely related to molecular weight of each compound and the individual lipid concentration (Le et al. 2014). In general, the relation between the hydrocarbon concentrations in the parasite *O. wageneri* and in the fish *C. chittendeni* increased with increasing K_ow_ and the number of rings. Previous studies have shown that lipid concentrations in the parasites (primarily intestinal endoparasites) are usually lower than the corresponding concentrations in their hosts (Le et al. 2014). Therefore, because they have lower overall lipid contents, the accumulation of lipophilic substances is expected to be lower in parasites than in the host tissues. However, our study found the opposite pattern—higher lipophilic concentrations in parasite tissues. This is likely a result of the fact that (1) lipid concentrations found in *O. wageneri* apparently were enough to accumulate PAHm, at least in concentrations higher than its host *C. chittendeni* or (2) the partition behavior of PAHm in this parasite–host system was different than for other organic pollutants (e.g., polychlorinated biphenyls) (Heinonen et al. 1999; Persson et al. 2007; Le et al. 2014). Interestingly, Heinonen et al. (2001) found an increased survival and lethal body burden of infected compared with uninfected bivalves exposed to pentachlorophenol (PCP). They suggested that these differences may be associated with the higher lipid content found in parasites which could change the internal distribution of PCP. Additionally, Yang et al. (2009) explain that cestodes cannot synthesize long-chain fatty acids and they efficiently obtain lipids directly from their host’s intestinal lumen. Thus, cestodes probably absorb PAHm with the contents of the host intestinal lumen. Certainly, more intensive laboratory work is necessary to define the behavior of chemical fractionation of PAHm in host–parasite systems.

Our results also showed a spatial trend of increasing parasite number and decreasing parasite PAHm concentration with increase in the distance from the oil wells. Several studies have reported that, in general, concentrations of hydrocarbons, barium, and trace metals decrease with increasing distance from the drill sites, with positive effect for benthic communities. For example, Ellis et al. (2012) documented the change in benthic communities, including loss of biodiversity and a stop in feeding behavior owing to the proximity to oil exploration and extraction activities. Because PAHm in *O. wageneri* were higher than those in their hosts, our results suggest that this type of biomarker would provide more precise information on the exposure of both parasites and their host to pollutants than effect indicators, such as changes in metrics at population or community levels, because the latter are influenced by other environmental conditions over time.

## Conclusions

This study showed that the larval cestode, *O. wageneri*, was able to accumulate PAHm in higher concentrations than its host, the Mexican flounder *C. chittendeni*. Apparently, the reason for this difference was that the fish has greater capacity to eliminate PAHm than the parasite. Under these circumstances, this parasite–host system *O. wageneri* could be considered as a better indicator of PAHm bioavailable concentrations in oil extraction zones than its fish host. Based on the results presented here, it is unclear whether *O. wageneri* is able to produce its own PAHm metabolites or if it simply acquires them from its host. Certainly, laboratory experiments are required to address this issue.

## Electronic supplementary material

ESM 1 (DOCX 170 kb)

## Data Availability

The datasets generated during and/or analyzed during the current study are available from the corresponding author upon reasonable request.
