# Supplementary material for: Polycyclic aromatic hydrocarbons in the cestode Oncomegas wageneri parasite of Mexican flounder Cyclopsetta chittendeni
Source: Parasitol Res. 2020 Feb 1;119(3):903–13. doi: 10.1007/s00436-019-06597-z (PMC7075845; doi:10.1007/s00436-019-06597-z)
Supplement: Supplementary file 1 — (DOCX 170 kb) [file 436_2019_6597_MOESM1_ESM.docx]

**Supplementary material**

**Table S1.** ANOVA regression test to assess the homogeneity of variances for Benzo[a]pyrene.

| **Regression statistics** | |
| --- | --- |
| Multiple correlation coefficient | 0.999 |
| Determination coefficient | 0.999 |
| R^2^ set | 0.999 |
| Typical error | 4.526 |
| Remarks | 6 |

| **Statistical Variance Analysis** | | | | | |
| --- | --- | --- | --- | --- | --- |
|  | Degrees of freedom | Sum of squares | Average of squares | F | Critical value of F |
| Regression | 1 | 106860508.510 | 106860508.510 | 5216367.350 | 2.205E-13 |
| Residues | 4 | 81.940 | 20.490 |  |  |
| Total | 5 | 106860590.450 |  |  |  |

|  | **Coefficients** | **Typical error** | **Statistical t** | **Probability** | **Lower 95%** | **High 95%** |
| --- | --- | --- | --- | --- | --- | --- |
| Intercept | -2.374 | 3.283 | -0.723 | 0.510 | -11.489 | 6.742 |
| Variable X 1 | 3971.407 | 1.739 | 2283.937 | 0.000 | 3966.579 | 3976.235 |

**Table S2.** ANOVA regression test to assess the homogeneity of variances for 1-Hydroxypyrene.

| **Regression statistics** | |
| --- | --- |
| Multiple correlation coefficient | 0.999 |
| Determination coefficient | 0.999 |
| R^2^ set | 0.999 |
| Typical error | 3.168 |
| Remarks | 6 |

| **Statistical Variance Analysis** | | | | | |
| --- | --- | --- | --- | --- | --- |
|  | Degrees of freedom | Sum of squares | Average of squares | F | Critical value of F |
| Regression | 1 | 302588699.600 | 302588699.600 | 30144463.350 | 6.603E-15 |
| Residues | 4 | 40.150 | 10.040 |  |  |
| Total | 5 | 302588739.750 |  |  |  |

|  | **Coefficients** | **Typical error** | **Statistical t** | **Probability** | **Lower 95%** | **High 95%** |
| --- | --- | --- | --- | --- | --- | --- |
| Intercept | -1.662 | 2.298 | -0.723 | 0.510 | -8.042 | 4.719 |
| Variable X 1 | 7654.909 | 1.394 | 5490.397 | 0.000 | 7651.038 | 7658.780 |

**Table S3.** ANOVA regression test to assess the homogeneity of variances for Phenanthrene.

| **Regression statistics** | |
| --- | --- |
| Multiple correlation coefficient | 0.999 |
| Determination coefficient | 0.999 |
| R^2^ set | 0.999 |
| Typical error | 34.638 |
| Remarks | 6 |

| **Statistical Variance Analysis** | | | | | |
| --- | --- | --- | --- | --- | --- |
|  | Degrees of Freedom | Sum of squares | Average of squares | F | Critical value of F |
| Regression | 1 | 261048160.630 | 261048160.630 | 217567.940 | 1.300E-10 |
| Residues | 4 | 4799.390 | 1199.850 |  |  |
| Total | 5 | 261052960.020 |  |  |  |

|  | **Coefficients** | **Typical error** | **Statistical t** | **Probability** | **Lower 95%** | **High 95%** |
| --- | --- | --- | --- | --- | --- | --- |
| Intercept | 31.710 | 25.126 | 1.262 | 0.275 | -38.052 | 101.472 |
| Variable X 1 | 87.877 | 0.188 | 466.442 | 0.000 | 87.354 | 88.400 |

**Table S4.** ANOVA regression test to assess the homogeneity of variances to 2-Naphthol.

| **Regression statistics** | |
| --- | --- |
| Multiple correlation coefficient | 0.999 |
| Determination coefficient | 0.999 |
| R^2^ set | 0.999 |
| Typical error | 34.589 |
| Remarks | 6 |

| **Statistical Variance Analysis** | | | | | |
| --- | --- | --- | --- | --- | --- |
|  | Degrees of freedom | Sum of squares | Average of squares | F | Critical value of F |
| Regression | 1 | 50199934.850 | 50199934.900 | 41959.840 | 3.400E-09 |
| Residues | 4 | 4785.520 | 1196.380 |  |  |
| Total | 5 | 50204720.370 |  |  |  |

|  | **Coefficients** | **Typical error** | **Statistical t** | **Probability** | **Lower 95%** | **High 95%** |
| --- | --- | --- | --- | --- | --- | --- |
| Intercept | -19.047 | 25.090 | -0.759 | 0.490 | -88.708 | 50.614 |
| Variable X 1 | 47.635 | 0.232 | 204.841 | 0.000 | 46.989 | 48.281 |

**Table S5.** Percentage of recovery (Accuracy) and the relative standard deviation (Precision) for Benzo[a]pyrene.

| **Replicate** | **Level 1** | **Level 2** | **Level 3** |
| --- | --- | --- | --- |
| **Precision** | | | |
|  | 0.628 ng/mL | 1.876 ng/mL | 3.111 ng/mL |
| 1 | 0.619 | 1.960 | 3.099 |
| 2 | 0.620 | 1.867 | 3.102 |
| 3 | 0.620 | 1.867 | 3.102 |
| 4 | 0.600 | 1.845 | 3.145 |
| 5 | 0.546 | 1.671 | 3.129 |
| 6 | 0.683 | 1.741 | 3.009 |
| Standard deviation (SD) | 0.044 | 0.103 | 0.047 |
| Mean | 0.615 | 1.825 | 3.098 |
| Relative standard deviation (% rsd) | 7.200 | 5.600 | 1.500 |
| **Accuracy** | | | |
|  | 0.628 ng/mL | 1.876 ng/mL | 3.111 ng/mL |
| 1 | 99.000 | 104.500 | 99.600 |
| 2 | 99.200 | 99.500 | 99.700 |
| 3 | 99.200 | 99.500 | 99.700 |
| 4 | 96.000 | 98.300 | 101.100 |
| 5 | 87.400 | 89.100 | 100.600 |
| 6 | 109.400 | 92.800 | 96.700 |
| **Mean** | **98.300** | **97.300** | **99.600** |

**Table S6.** Percentage of recovery (Accuracy) and the relative standard deviation (Precision) for Hydroxypyrene.

| **Replicate** | **Level 1** | **Level 2** | **Level 3** |
| --- | --- | --- | --- |
| **Precision** | | | |
|  | 0.549 ng/mL | 1.638 ng/mL | 2.716 ng/mL |
| 1 | 0.547 | 1.619 | 2.776 |
| 2 | 0.524 | 1.631 | 2.758 |
| 3 | 0.528 | 1.597 | 2.807 |
| 4 | 0.546 | 1.603 | 2.771 |
| 5 | 0.629 | 1.718 | 2.796 |
| 6 | 0.629 | 1.718 | 2.796 |
| Standard deviation (SD) | 0.049 | 0.056 | 0.018 |
| Mean | 0.567 | 1.648 | 2.784 |
| Relative standard deviation (% rsd) | 8.600 | 3.400 | 0.700 |
| **Accuracy** | | | |
|  | 0.549 ng/mL | 1.638 ng/mL | 2.716 ng/mL |
| 1 | 100.000 | 98.900 | 102.200 |
| 2 | 95.900 | 99.600 | 101.500 |
| 3 | 96.500 | 97.500 | 103.300 |
| 4 | 99.800 | 97.900 | 102.000 |
| 5 | 114.900 | 104.900 | 103.000 |
| 6 | 114.900 | 104.900 | 103.000 |
| **Mean** | **103.600** | **100.600** | **102.500** |

**Table S7.** Percentage of recovery (Accuracy) and the relative standard deviation (Precision) for Phenanthrene.

| **Replicate** | **Level 1** | **Level 2** | **Level 3** |
| --- | --- | --- | --- |
| **Precision** | | | |
|  | 44.389 ng/mL | 132.506 ng/mL | 219.753 ng/mL |
| 1 | 42.819 | 145.130 | 218.330 |
| 2 | 44.587 | 119.850 | 237.074 |
| 3 | 38.622 | 126.099 | 226.257 |
| 4 | 45.029 | 117.767 | 219.081 |
| 5 | 43.557 | 131.533 | 218.640 |
| 6 | 44.574 | 131.745 | 219.081 |
| Standard deviation (SD) | 2.383 | 9.918 | 7.486 |
| Mean | 43.198 | 128.687 | 223.077 |
| Relative standard deviation (% rsd) | 5.500 | 7.700 | 3.400 |
| **Accuracy** | | | |
|  | 44.389 ng/mL | 132.506 ng/mL | 219.753 ng/mL |
| 1 | 96.500 | 109.500 | 99.400 |
| 2 | 100.400 | 90.400 | 107.900 |
| 3 | 87.000 | 95.200 | 103.000 |
| 4 | 101.400 | 88.900 | 99.700 |
| 5 | 98.100 | 99.300 | 99.500 |
| 6 | 100.400 | 99.400 | 99.700 |
| **Mean** | **97.300** | **97.100** | **101.500** |

**Table S8.** Percentage of recovery (Accuracy) and the relative standard deviation (Precision) for Naphtol.

| **Replicate** | **Level 1** | **Level 2** | **Level 3** |
| --- | --- | --- | --- |
| **Precision** | | | |
|  | 35.910 ng/mL | 107.196 ng/mL | 177.778 ng/mL |
| 1 | 35.862 | 105.507 | 181.162 |
| 2 | 32.657 | 116.473 | 166.452 |
| 3 | 35.640 | 103.397 | 177.323 |
| 4 | 33.277 | 97.484 | 189.461 |
| 5 | 44.971 | 116.257 | 186.840 |
| 6 | 44.971 | 116.257 | 188.957 |
| Standard deviation (SD) | 5.623 | 8.210 | 8.846 |
| Mean | 37.896 | 109.229 | 181.699 |
| Relative standard deviation (% rsd) | 14.800 | 7.500 | 4.900 |
| **Accuracy** | | | |
|  | 35.910 ng/mL | 107.196 ng/mL | 177.778 ng/mL |
| 1 | 99.900 | 98.400 | 101.900 |
| 2 | 90.900 | 108.700 | 93.600 |
| 3 | 99.200 | 96.500 | 99.700 |
| 4 | 92.700 | 90.900 | 106.600 |
| 5 | 125.200 | 108.500 | 105.100 |
| 6 | 125.200 | 108.500 | 106.300 |
| **Mean** | **105.500** | **101.900** | **102.200** |

**Table S9.** Values of the Akaike information criterion (AIC) of the distributions tested for the Generalized Lineal Model (GLM). Parasite PAH metabolite concentrations were used as dependent variable and the independent variables were number of individual parasites, proximity to oil wells, BCF and host total PAH concentrations.

| **Distribution** | **Akaike criterion (AIC)** |
| --- | --- |
| Gaussian | 148.20 |
| Gamma | 150.64 |
| Inverse Gaussian | 152.39 |





**Figure S1.** Calibration curves for aromatic hydrocarbon metabolites measured.
